# The impact of vasomotion on analysis of rodent fMRI data

**DOI:** 10.3389/fnins.2023.1064000

**Published:** 2023-02-24

**Authors:** Henriette Lambers, Lydia Wachsmuth, Chris Lippe, Cornelius Faber

**Affiliations:** Clinic of Radiology, University of Münster, Münster, Germany

**Keywords:** BOLD fMRI, vasomotion, hemodynamic oscillations, small animals, anesthesia, GLM, brain networks

## Abstract

**Introduction:**

Small animal fMRI is an essential part of translational research in the cognitive neurosciences. Due to small dimensions and animal physiology preclinical fMRI is prone to artifacts that may lead to misinterpretation of the data. To reach unbiased translational conclusions, it is, therefore, crucial to identify potential sources of experimental noise and to develop correction methods for contributions that cannot be avoided such as physiological noise. Aim of this study was to assess origin and prevalence of hemodynamic oscillations (HDO) in preclinical fMRI in rat, as well as their impact on data analysis.

**Methods:**

Following the development of algorithms for HDO detection and suppression, HDO prevalence in fMRI measurements was investigated for different anesthetic regimens, comprising isoflurane and medetomidine, and for both gradient echo and spin echo fMRI sequences. In addition to assessing the effect of vasodilation on HDO, it was studied if HDO have a direct neuronal correlate using local field potential (LFP) recordings. Finally, the impact of HDO on analysis of fMRI data was assessed, studying both the impact on calculation of activation maps as well as the impact on brain network analysis. Overall, 303 fMRI measurements and 32 LFP recordings were performed in 71 rats.

**Results:**

In total, 62% of the fMRI measurements showed HDO with a frequency of (0.20 ± 0.02) Hz. This frequent occurrence indicated that HDO cannot be generally neglected in fMRI experiments. Using the developed algorithms, HDO were detected with a specificity of 95%, and removed efficiently from the signal time courses. HDO occurred brain-wide under vasoconstrictive conditions in both small and large blood vessels. Vasodilation immediately interrupted HDO, which, however, returned within 1 h under vasoconstrictive conditions. No direct neuronal correlate of HDO was observed in LFP recordings. HDO significantly impacted analysis of fMRI data, leading to altered cluster sizes and *F*-values for activated voxels, as well as altered brain networks, when comparing data with and without HDO.

**Discussion:**

We therefore conclude that HDO are caused by vasomotion under certain anesthetic conditions and should be corrected during fMRI data analysis to avoid bias.

## 1. Introduction

Functional magnetic resonance imaging (fMRI) allows for non-invasive mapping of neuronal activity as well as investigation of functional connectivity. However, caution must be taken with interpretation of fMRI, as it is an indirect representation of neuronal activity. fMRI is based on blood oxygenation level dependent (BOLD) contrast (Ogawa et al., 1990; Buxton, 2013), as the hemodynamic response to neuronal activity causes changes in the MR signal. Therefore, in addition to technical noise and motion, physiological noise including heartbeat, respiration, arterial carbon dioxide (CO_2_) concentration and vasomotion can distort the functional signal (Murphy et al., 2013). This is crucial for functional network analysis, since any non-neuronal related process that affects at least one brain region impacts analysis of functional connectivity. Furthermore, non-neuronal signals may affect calculation of BOLD activation maps, since models used for fMRI data analysis with the general linear model (GLM) usually ignore physiological noise. Especially in small animal fMRI, physiological noise must be studied carefully, since measurements are often acquired under anesthesia, which alter physiological processes (Gao et al., 2017; Paasonen et al., 2018; van Alst et al., 2019) compared to the awake condition. Small animal fMRI is valuable for translational research, since it offers numerous options such as studying specific disease models (Tenney et al., 2003; Tristao Pereira et al., 2021; Wachsmuth et al., 2021), pharmacological (Haensel et al., 2015) and surgical interventions (Chen et al., 2019) as well as combining MRI with electrophysiological (Pan et al., 2010; Kosten et al., 2022) and optical recordings (Schulz et al., 2012; Albers et al., 2018; Lake et al., 2020; Ioanas et al., 2022). To avoid incorrect translational conclusions, it is crucial to investigate physiological noise and to develop correction methods for commonly performed preclinical experiments.

Although fMRI can be conducted on awake animals, small animal fMRI studies are often performed under anesthesia to reduce motion and prevent stress for the animal. A variety of anesthetic drugs is used for fMRI studies, including α-chloralose, isoflurane (ISO), ketamine-xylazine, propofol, urethane or medetomidine/dexmedetomidine (MED/DMED). Frequently, modifications of an anesthetic protocol originally proposed by Weber et al. (2006) are used, whereby animal preparation is performed under an inhalation anesthetic followed by fMRI measurements under MED sedation. This protocol offers a number of advantages, since application is non-invasive, can be maintained over extended durations and leads to temporally stable neuronal activity and functional connectivity in rats (Weber et al., 2006; Sirmpilatze et al., 2019). Using a similar anesthesia protocol (animal preparation under ISO, fMRI under DMED), Magnuson et al. (2010, 2014) detected signal oscillations with a frequency of approximately 0.2 Hz in CBV and in BOLD weighted fMRI measurements. The authors hypothesized that these oscillations resulted either from infraslow neuronal activity or from modulations in vascular tone. Close examination of these oscillations is important, because they may represent a physiological noise source that distorts fMRI data analysis. We refer to these oscillations in the following as hemodynamic oscillations (HDO), since both neuronal activity and vascular tone model the hemodynamic system.

**FIGURE 1:**
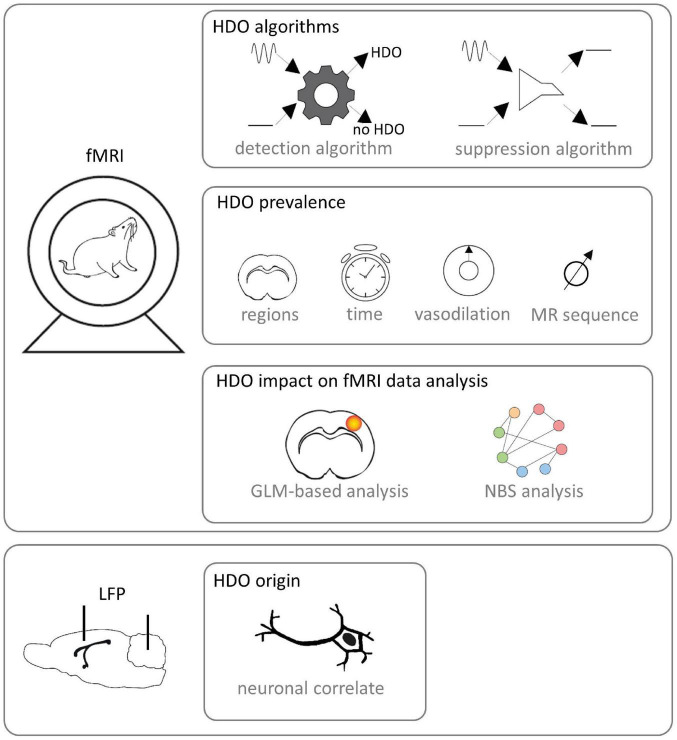
Study design. fMRI was performed on Fischer rats. Algorithms for detection and suppression of HDO were developed. Afterward, HDO prevalence was investigated using the detection algorithm. In detail, it was examined in which brain regions and after which time HDO occurred after switching from ISO anesthesia to MED sedation. Further, the effect of vasodilation was studied and it was examined whether HDO occur in both SE-EPI and GE-EPI scans. Subsequently, the impact of HDO to GLM-based analysis and NBS analysis of fMRI data was examined using the developed algorithms. Additionally, LFP signals were recorded with implanted electrodes for examination whether HDO have a direct neuronal correlate.

This study aimed to investigate origin and prevalence of HDO in fMRI as well as their impact on data analysis (Figure 1). For this purpose, we performed fMRI with two different MR contrasts under ISO and MED anesthesia. We developed algorithms for detection and suppression of HDO, and investigated the effect of vasodilation on HDO. Further, we studied whether HDO are physiological noise or whether they have a direct neuronal correlate, using local field potential (LFP) recordings. Finally, we investigated the impact of HDO on fMRI data analysis using the GLM (Friston et al., 1995) as well as brain network analysis using network based statistics (NBS) (Zalesky et al., 2010).

## 2. Materials and methods

All experiments were conducted according to the German Animal Welfare Act and were approved by the State Agency for Nature, Environment and Consumer Protection of North Rhine-Westphalia, Germany (LANUV approval IDs: 87-51.04.2010.A274, 84-02.04.2015.A427, 84-02.04.2016.A135, and 81-02.04.2018.A426). Experiments were performed with 71 adult Fischer rats (>3 month). A total of 70 animals were female and had a weight of (184 ± 14) g, one animal was male and weighed 261 g. Rats were housed in groups of two to five animals under a regular light/dark schedule (12/12 h) with food and water *ad libitum*.

### 2.1. Animal handling and data overview

Respiratory gas was 1 L/min O_2_ for anesthesia induction and during LFP experiments. For MRI, a gas mixture of 0.25 L/min O_2_ and 0.75 L/min air was used. Animal preparation and LFP electrode implantations were performed under ISO anesthesia (5% induction, 2–3% maintenance). Long-term ISO experiments were performed under 1.2% ISO anesthesia. Other experiments were executed after switching to MED sedation initiated by a subcutaneous bolus injection of 0.04 mg/kg followed by a continuous infusion of 0.05 mg/kg/h. After MED bolus, ISO was discontinued within 10–15 min and experiments were started at least 40 min after bolus injection, because after this waiting period a stable physiology can be expected (Weber et al., 2006; Sommers et al., 2009; Amirmohseni et al., 2016; Sirmpilatze et al., 2019; Kint et al., 2020). A total of 30 min prior to stereotaxic surgery for electrode implantation, the animals received analgesia [Metacam (1 mg/kg s.c.) or Metamizol (100 mg/kg s.c.)]. A total of 56 animals were ventilated (MRI-1 Ventilator, CWE, Inc., Ardmore, Pennsylvania, United States). A total of 24 of them received a muscle relaxant [Pancuronium (2 mg/kg bolus followed by continuous injection of 1.5 mg/kg/h) or Atracurium (5 mg/kg bolus followed by continuous injection of 5 mg/kg/h)]. For ventilated animals, the end-expiratory CO_2_ was continuously monitored using a CO_2_ analyzer (Micro CapStar End-Tidal CO_2_ Analyzer, CWE, Inc., Ardmore, Pennsylvania, United States). Respiration rate of ventilated animals was set to 53, 57, or 60 breaths per minute (bpm). The respiratory minute volume was between 95 and 127 mL/min. Respiration rate of spontaneously breathing animals was (56 ± 5) bpm and (68 ± 11) bpm for LFP and fMRI, respectively. Animals were placed on a temperature-controlled bed and fixated with bite and ear bars. Rectal temperature was kept at (36.7 ± 0.4) °C. Functional echo-planar imaging (EPI) and LFP data from seven experiments were investigated: (1) For up to 4 h, 5 or 10 min-long gradient echo-EPI (GE-EPI) measurements were performed repeatedly under 1.2% ISO anesthesia. (2) For up to 8 h short GE-EPI scans were acquired repeatedly under MED sedation. (3) Spin echo-EPI (SE-EPI) measurements were recorded under MED sedation. GE-EPI scans were recorded during execution of (4) CO_2_ or (5) ISO challenges. (6) GE-EPI measurements were recorded during electrical paw stimulation. (7) LFP recordings were conducted. A total of 10% of the data were included retrospectively from previous studies (Albers et al., 2019; van Alst et al., 2019; Lambers et al., 2022). Therefore, temporal resolution of MR measurements varied. For characterization of oscillation frequency, optimization and evaluation of the oscillation detection algorithm, a training and test dataset was generated: For both training and test data, 240 time courses each were randomly selected from 2,254 time courses of high temporal resolution GE-EPI data. As a previous study (Lambers et al., 2020) implied that hemodynamics were not gender-specific, measurements of both sexes were combined. Grouping information is provided in Supplementary Table 1.

### 2.2. Electrical paw stimulation and vasodilation challenges

For electrical paw stimulation, two electrodes were inserted into one forepaw and 1-ms pulses were applied at a frequency of 9 Hz with an amplitude of 1 or 1.5 mA according to block paradigms. Further details are given in the respective figure legends. Approximately 50% of GE-EPI scans used for algorithm optimization and investigation of oscillation prevalence were recorded during electrical paw stimulation, the other 50% were recorded without stimulation (resting state). However, both resting state and stimulation fMRI data were pooled for analysis. To examine the effect of HDO on analysis of fMRI data, stimulation was applied with the following paradigm: 5-s stimulation, 25-s rest, 20 repetitions. For CO_2_ challenge experiments, the mixture of inspiratory gas was alternately set to standard (0.25 L/min O_2_, 0.75 L/min air, and 0.00 L/min CO_2_) and 5% CO_2_ (0.20 L/min O_2_, 0.75 L/min air, and 0.05 L/min CO_2_) according to the following paradigm: 3-min standard, 3-min 5% CO_2_, 4-min standard. All animals undergoing CO_2_ challenge were ventilated to enable recording of CO_2_ levels in expiratory air. Starting point of the CO_2_ challenge was defined at the moment when the expired CO_2_ level exceeded baseline by more than 50%. End point of the CO_2_ challenge was defined as the moment when the expired CO_2_ level fell below 50% above baseline. Only datasets were evaluated in which the CO_2_ challenge was at least 2.5 min long. Additionally, ISO challenge experiments were conducted using the following paradigm: 5-min no ISO, 10-min ISO (0.8, 1.5, or 2%), 10-min no ISO.

### 2.3. Functional magnetic resonance imaging experiments

MR measurements were acquired using a 9.4 T Bruker Biospec 94/20 small animal scanner equipped with a 720 mT/m gradient system (Bruker BioSpin GmbH, Ettlingen, Germany) and different combinations of transmit coils and receive-only coils. First, an anatomical image was acquired to identify the slice position for fMRI measurements: 2D Turbo spin echo sequence (RARE), TR/TE_*Eff*_ = 2,000/50 ms, RARE factor 8, Matrix 256 × 256, field of view (FOV) 28 mm × 26 mm, slice thickness 1.2 mm, 9–12 contiguous slices. Subsequently, B_0_ homogenization was performed using the MAPSHIM Bruker routine. Functional GE-EPI and SE-EPI measurements were performed using a single-shot EPI sequence (same FOV as anatomy, Matrix: 80 × 80, 1.2 mm slice thickness; GE-EPI: TE 18 ms, TR 100/125/1,000 ms, flip angle 18/21/60 or 65°, 1/2/9 or 12 slices; SE-EPI: TE 35.9 ms, TR 250 ms, flip angle 90°, 3 slices). One fMRI measurement lasted between 5 and 25 min (Supplementary Table 1).

### 2.4. Functional magnetic resonance imaging data processing

This section describes the processing of all fMRI data with exception of the data analyzed using the GLM or NBS (see Section “2.5. Functional magnetic resonance imaging data analysis using the GLM or NBS”). Data processing was done using MATLAB (Release 2021b, The MathWorks, Inc., Natick, Massachusetts, United States). The first 5 s of each measurement were discarded to avoid pre-steady-state artifacts. A template of 14 regions based on a rat brain atlas (Paxinos, 1997) was registered to the data (Supplementary Figure 1). If the measurements contained only one slice of the template, seven regions were registered. These regions were selected since they were not expected to contain strong susceptibility artifacts, which would affect comparison of SE-EPI and GE-EPI data. For vasodilation challenge experiments, only the right forelimb region of the primary somatosensory cortex (S1Fl) was examined. Signals from voxels located in one region were summed up, downsampled to a temporal resolution of 1 s and normalized to their mean. For determination of the oscillation frequency, no downsampling was performed, as a higher temporal resolution was required for the fitting procedure.

### 2.5. Functional magnetic resonance imaging data analysis using the GLM or NBS

Data analysis using both, GLM or NBS was performed on data recorded for 10 min during electrical paw stimulation. For preprocessing, the first five iterations were removed, data were realigned using SPM12^1^ and brains were manually masked using MRIcroGL^2^ or MagnAN (BioCom, Uttenreuth). Subsequently, analysis was performed using GLM or NBS. GLM-based analysis was done with SPM 12 by using the 3rd order canonical basis set: convolution of the stimulation paradigm and a rat HRF (Lambers et al., 2020) and its derivatives were used as regressors. For each dataset, two BOLD activation maps were calculated using an *F*-test (*p* < 0.05, family wise error correction): before and after voxel-wise application of the HDO suppression algorithm. Effect of HDO on GLM-based analysis was only examined on datasets with more than 12 and a maximum of 100 activated voxels in the activated S1Fl (before suppression algorithm application). Data were divided into non-oscillatory and oscillatory by analyzing time courses of the activated S1Fl with the HDO detection algorithm. The evaluation of the algorithm was reviewed using manual classifications. Two datasets were excluded from analysis due to large differences between manual and automated classification.

Additionally, NBS analysis was conducted with MagnAn. In most cases, stimulation was applied to the left paw. Scans were mirrored, if the right paw was stimulated. Data were smoothed (gaussian kernel, 3 × 3 pixel, full width half max 0.6 mm) and lowpass filtered at 0.3 Hz (Fourier filter). Subsequently, regression of the global mean signal was performed and a template of 28 brain regions based on the Paxinos and Watson rat brain atlas (Paxinos, 1997) was registered to the data (Supplementary Table 2). Non-registerable datasets (e.g., measurements that did not cover both hemispheres) were excluded from analysis. If an animal was measured twice, registration was done on the first measured scan. Data were divided into non-oscillatory and oscillatory by analyzing time courses of all 28 brain regions with the HDO detection algorithm. For each dataset, Pearson correlation coefficients were calculated between pairs of brain regions. Resulting 28 × 28 correlation matrices represented undirected, weighted functional connections between brain regions. Only positive correlation coefficients were considered. Per dataset, correlation matrices were calculated twice (before and after voxel-wise application of the HDO suppression algorithm). Subsequently, mean correlation coefficients were calculated group wise. For this purpose, Pearson’s *r*-values were converted to Fisher’s *z*-values to provide normal distribution, averaged, and converted back to Pearson’s *r*-values. Resulting connectivity matrices (Supplementary Figure 2) clearly showed interhemispheric connections, indicating that the analysis detected meaningful functional connections. Subsequently, for the 140 strongest connections that appeared in the averaged matrices, NBS was used to find differences in functional connectivity: a paired *t*-test was performed between correlation matrices of HDO-suppressed und unsuppressed data and the largest components of significantly modulated connections (*p* < 0.05) were retained. Subsequently, permutation testing was applied with 1,000 iterations for correcting the *t*-test for multiple testing. Finally, mean differences of correlation coefficients (MDCC) were calculated separately for oscillating and non-oscillating data. For this purpose, differences of correlation matrices (suppressed--unsuppressed) were calculated for each significantly different connection and averaged over datasets. For visualizing results of network analysis, the software Cytoscape was used.^3^

### 2.6. Hemodynamic oscillations frequency fitting

To determine the oscillation frequency for all 240 time courses of the test dataset, a Fast Fourier Transformation (FFT) was calculated. A total of 60 of the resulting spectra showed a peak between 0.08 and 0.8 Hz. Corresponding time courses were classified as oscillating and the oscillation frequency was determined. For this purpose, a function consisting of an exponential and a Gaussian term (exponential-Gaussian function, EGF) was fitted to the spectra across the interval from 0.01 to 1 Hz.


$$E\,G\,F\,\left(f\right)=a+b\cdot e^{-c\cdot f}+d\cdot e^{-\frac{{\left(f-f_{o}\right)}^{2}}{2\,\sigma^{2}}}.$$

The parameter *f_o_* represented the frequency of the HDO while σ was the width of the peak in the spectrum caused by the oscillations. Frequencies and peak widths determined using the test data were averaged and used for optimization of the detection algorithm and the suppression algorithm. Parameters from two EGFs were excluded from averaging since one of the fitted parameters was unphysiological (negative peak amplitude or peak position).

### 2.7. Hemodynamic oscillations detection and suppression algorithms

In order to systematically investigate the prevalence of HDO and their effect on the analysis of fMRI data, we developed two algorithms using MATLAB. Both require time courses extracted from fMRI data as input and are online available.^4^ The first algorithm detects oscillations and delivers the output whether the inserted time course contains HDO. The second algorithm suppresses HDOs from the inserted time course.

The HDO suppression algorithm performed double filtering of the time courses with a finite impulse response (FIR) bandstop filter. Using the averaged oscillation frequency *f_o_* and peak width σ the passband of the FIR filter was set to [*f*_*o*_−3σ,*f*_*o*_ + 3σ] while the interval of the stopband was set to [*f*_*o*_−σ,*f*_*o*_ + σ]. The filter attenuation was adaptively adjusted. For this purpose, the FFT of the time course was calculated, the interval [*f*_*o*_−4σ,*f*_*o*_ + 4σ] of the spectrum was extracted and downsampled to a spectral resolution of half the peak width σ. The ratio of maximum and baseline of the downsampled spectrum was used for filter attenuation.

The HDO detection algorithm examined the FFT of the inserted time courses as well as the autocorrelation functions, since both are different for oscillating data compared to non-oscillating time courses: The FFT of oscillating data showed a peak while the autocorrelation function showed a periodic pattern which were absent for non-oscillating data. The detection algorithm first calculated the FFT of the extracted MR time courses. The interval [*f*_*o*_−4σ,*f*_*o*_ + 4σ] was extracted from the resulting spectrum using the averaged oscillation frequency *f_o_* and peak width σ. Baseline drifts were removed from the extracted interval using the function “detrend” and the detrended spectrum was downsampled to a spectral resolution of half the peak width σ. Additionally, the autocorrelation function of the time course was calculated for 15 time lags between 1 and 15 s using the function “autocorr.” Baseline drifts of the autocorrelation function were corrected using the function “detrend.” Subsequently, the detrended autocorrelation was averaged by calculating the mean of points that were 5 s apart from each other. This was done because the averaged autocorrelations of oscillatory and non-oscillatory time courses differed: Values of averaged autocorrelation functions of non-oscillatory time courses were around zero. Values for oscillatory time courses were non-zero, since their autocorrelation showed a periodic pattern with a period of 5 s. Both the averaged autocorrelation function, and the averaged spectrum, were transferred to an artificial neural network (ANN), which classified the data as non-oscillatory or oscillatory. Training of the ANN was done using 226 manually classified time courses of the training dataset (240 time courses), separating oscillating from non-oscillating time courses. Subsequently, the performance of the ANN was evaluated using 231 manually classified time courses of the test dataset (240 separate time courses, Supplementary Figure 3). All MR time courses investigated in this study were screened for HDO using the trained ANN. The developed HDO detection algorithm can be applied to time courses extracted from MR data recorded with a TR of 1 s or less.

### 2.8. Local field potential recordings

For LFP recordings, two custom made silver electrodes (250 μm dimeter) were implanted in the brain and glued to the skull using dental cement. One electrode was positioned in the S1Fl. The other electrode was used as reference and implanted in the cerebellum. LFP signals were recorded as difference between electric currents detected by the electrode in the S1Fl and the reference electrode using a differential amplifier (DPA-2FX, NPI Electronics, Tamm, Germany). Signals were recorded at 2 kHz using a multifunction data acquisition device (PCIe-6363, National Instruments, Austin, Texas, United States) and a custom-written LabView script (National Instruments). LFP recordings were performed for 3 min during stimulation which evoked neuronal activation in S1Fl. Only LFP recordings with visible response to stimulation were evaluated. LFP data were normalized to their baseline and the FFT was calculated using MATLAB. Time courses and spectra were manually analyzed with respect to a neuronal correlate of HDO, as no HDO detection algorithm was available for LFP data.

### 2.9. Data representation

Results in the text are represented as mean ± standard deviation. Data clusters are displayed as overlay of bee swarm plots and boxplots. In boxplots, central marks represent the median, boxes include the 25th and 75th percentiles, whiskers extend to the last data points within the 1.5× interquartile and outliers are indicated as crosses. Spectra shown within one figure are scaled equally.

## 3. Results

**FIGURE 2:**
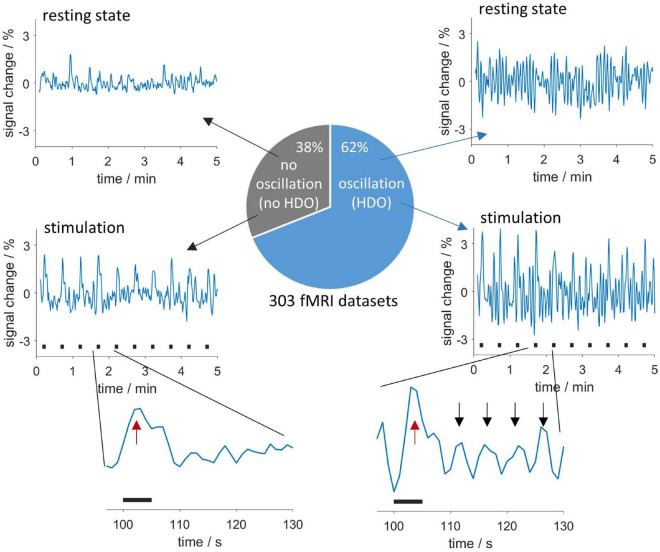
Appearance and occurrence of HDO. Periodic oscillations in fMRI time courses were investigated in 303 measurements. In total, 62% of the measurements showed HDO which had a period of approximately 5 s. Exemplary time courses without and with HDO for resting state data are shown in the **(upper row)** and data recorded during sensory stimulation in the **(middle row)**. Stimulation periods are indicated by black bars. Enlarged segments of one paradigm repetition **(bottom row)** showed that HDO (maxima indicated by black arrows) were triggered by the BOLD response (red arrows).

We investigated HDO, periodic oscillations with a period of approximately 5 s which were detected in signal time courses of fMRI measurements of anesthetized rats. These oscillations may represent a physiological source of noise for functional imaging. In total, 62% of all 303 fMRI measurements analyzed in this study showed HDO (Figure 2). For detailed investigation, we developed algorithms for detecting and suppressing HDO. Using these algorithms, we investigated HDO prevalence and assessed their impact on analysis of fMRI data.

### 3.1. Hemodynamic oscillations were detected at 0.2 Hz

**FIGURE 3:**
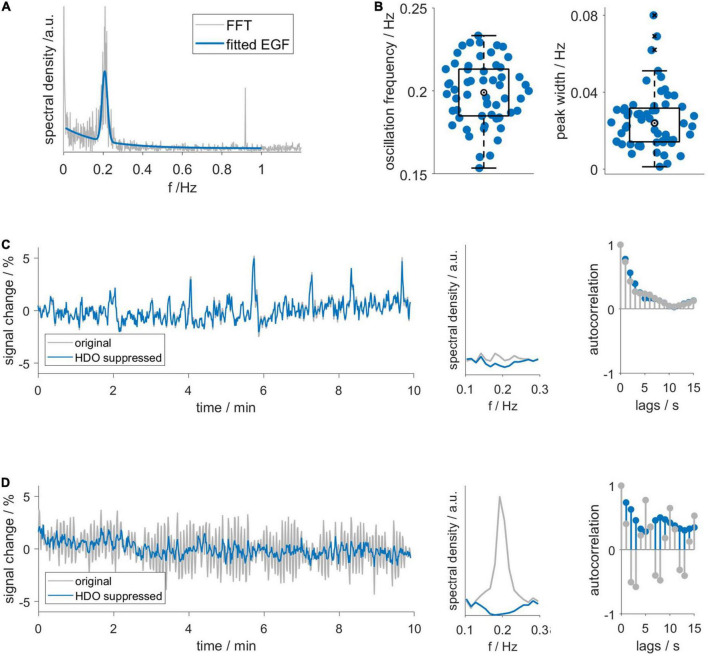
Hemodynamic oscillations frequency and assessment of suppression algorithm performance. **(A)** An exponential-gaussian function (EGF, blue) was fitted to FFT (gray) of an exemplary GE-EPI time course. Note that respiration caused a peak at 0.9 Hz. **(B)** HDO frequency *f_o_* and peak width σ, resulting from fitting 58 spectra. Representative **(C)** non-oscillatory and **(D)** oscillatory datasets: time courses (left), spectra (middle) and autocorrelation functions (right) before (gray) and after (blue) application of the HDO suppression algorithm. In contrast to the non-oscillatory dataset, the spectrum of the oscillating dataset showed a peak and the autocorrelation function showed a periodic pattern. The suppression algorithm eliminated HDO without substantially affecting non-oscillating signals.

First, we investigated the frequency domain of MR data using FFT and continuous wavelet transformation (CWT, Supplementary Figure 4). Spectra of datasets with HDO showed increased spectral density around 0.2 Hz. Additionally, respiration often caused a peak at approximately 1 Hz, yet this peak was not related to the occurrence of HDO. No other conspicuous peaks occurred in the spectra, suggesting that HDO were not directly driven by cardiac, respiratory, myogenic, neurogenic or endothelial oscillators. We characterized the HDO frequency using a fitting procedure with an EGF (Figure 3A). An HDO frequency *f_o_* of (0.20 ± 0.02) Hz with an average peak width σ of (0.025 ± 0.015) Hz was obtained (Figure 3B). Both HDO detection and suppression algorithms were adjusted using the determined frequency and peak width. Subsequently, the HDO suppression algorithm eliminated HDO without substantially affecting other signal components as exemplary shown in Figures 3C, D. Finally, the neural network of the detection algorithm was trained using the manually classified training data. After training, the algorithm performance was evaluated using the manually classified test data. The detection algorithm had a specificity of 95% (Supplementary Figure 3).

### 3.2. Vasodilation interrupted hemodynamic oscillations

**FIGURE 4:**
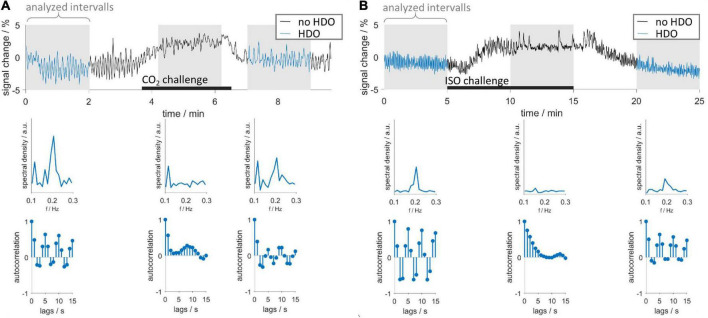
Vasodilation interrupts HDO. Vasodilation was induced by administration of **(A)** CO_2_ and **(B)** ISO. First row: exemplary time courses recorded during CO_2_ and ISO challenge. Three segments were analyzed using the HDO detection algorithm (gray background): before, during and after the respective challenge (black bars). Time course segments classified as oscillating were colored blue. Second and third row show spectra and autocorrelation functions, respectively, of the corresponding sections above.

CO_2_ and ISO challenges were used to investigate whether short-time vasodilation interrupts HDO. Time courses were extracted from S1Fl, and three intervals (before, during and after challenge) were examined (Figure 4). In 15 vasodilation challenge scans (CO_2_: *n* = 6, ISO: *n* = 9), time intervals preceding the challenge showed HDO. In all time courses, the MR signal increased during the challenge and subsequently returned to baseline, indicating that the challenge caused vasodilation. No HDO were detected during challenge in any of these measurements. We therefore concluded that vasodilation interrupted HDO. In 50% of CO_2_ challenge measurements, oscillations returned immediately after end of challenge. In 90% of ISO challenge scans, oscillations were detected 5 min after challenge had ended. Furthermore, it was investigated whether HDO occur during long-term ISO anesthesia, which is known to cause permanent vasodilation. Ten animals were measured for up to 4 h under ISO anesthesia without MED. From these, in a total of 56 GE-EPI scans 14 different brain regions were examined: Only 14 of the resulting 784 region-specific time courses were classified as oscillatory by the detection algorithm. This corresponded to 1.8%, which is considerably below the false positive rate of the detection algorithm. Thus, we concluded that the occurrence of HDO under long-term ISO anesthesia, i.e., permanent vasodilation, was negligible. In an analysis, described in Supplementary Figure 5, we found that short-term vasodilation induced by stimulation of neuronal activation also interrupted HDO briefly, resulting in HDO being stimulus-locked. This effect is consistent with two studies by Sirotin and Das (2009) and Sirotin et al. (2012), which reported stimulation-locked oscillations in hemodynamic signals.

### 3.3. Brain-wide hemodynamic oscillations started within 1 h after end of ISO anesthesia

**FIGURE 5:**
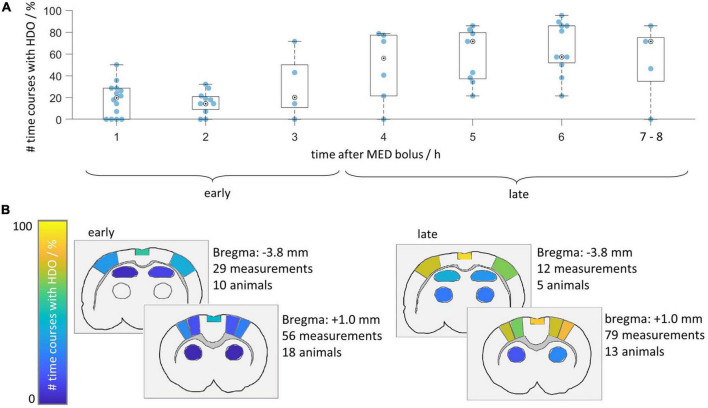
Hemodynamic oscillations prevalence increases over time. HDO prevalence was assessed in 135 GE-EPI scans of 23 animals measured after switching from ISO to MED anesthesia. **(A)** Time line illustrating relative prevalence of HDO. For each animal, all measurements acquired within 1 h were combined and the percentage of oscillating signal time courses was plotted. Not every animal was measured at all time points. Measurements recorded after more than 6 h were displayed together. **(B)** HDO prevalence maps separated into early (recorded 1–3 h after MED start) and late (recorded 4 h or later) measurements. Color codes relative number of scans with HDO in each of the 14 brain regions.

**FIGURE 6:**
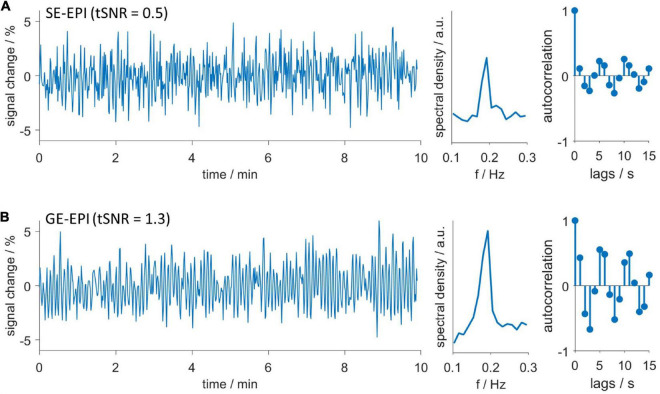
Hemodynamic oscillations are detected with both GE-EPI and SE-EPI. Exemplary oscillating **(A)** SE-EPI and **(B)** GE-EPI scans. Time courses (left), spectra (middle) and autocorrelation functions (right). Both datasets were recorded in the same animal consecutively without delay. Despite lower temporal signal to noise ratio (tSNR) of SE-EPI scans, oscillations were similarly detected in both SE-EPI and GE-EPI scans.

Next, we investigated after which duration HDO started when switching from ISO anesthesia to MED sedation. We further assessed whether HDO prevalence changed with prolonged MED sedation. For this purpose, the HDO prevalence in 135 GE-EPI scans from 23 animals over a scan time period of maximum 8 h was examined. In 85% of the measurements, at least one time course of the investigated brain regions was oscillatory. Data acquisition started approximately 1 h after switching anesthesia. At this time, ten of 14 animals showed HDO. HDO prevalence increased over time (Figure 5A): Around 20% of early recorded (1–3 h after switching anesthesia) time courses oscillated. For late measurements (4 h or more), the portion of oscillating time courses increased to approximately 50%. HDO prevalence maps showed that HDO occurred in all examined regions (Figure 5B). After assessing HDO prevalence using GE-EPI measurements, HDO occurrence in SE-EPI data was investigated. HDO were detected in both GE-EPI and SE-EPI scans (Figure 6). To assess HDO prevalence in SE-EPI measurements, 27 SE-EPI scans from seven animals were examined. SE-EPI scans of five animals showed HDO. In total, at least one oscillating time course was found in 63% of the SE-EPI measurements. In SE-EPI data, HDO were observed only in cortical regions (Supplementary Figure 6).

### 3.4. Hemodynamic oscillations significantly impact analysis of fMRI data

**FIGURE 7:**
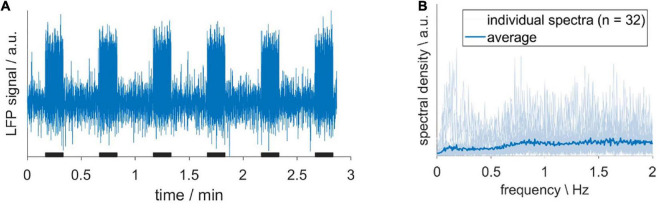
Hemodynamic oscillations do not have a direct neuronal correlate. **(A)** Exemplary LFP recording, acquired upon sensory stimulation with 10-s long stimulation periods (black bars). **(B)** Individual spectra (light blue) and their average (dark blue). The absence of a prominent peak around 0.2 Hz indicated that no oscillations were present.

Before we evaluated the impact of HDO on fMRI analysis, we assessed whether HDO are directly driven by neuronal activity. To this end, LFP recordings were performed in S1Fl, and scrutinized for electric activity patterns with frequencies around 0.2 Hz. A total of 32 LFP recordings from six animals were examined. The absence of a prominent peak around 0.2 Hz in the spectra indicated that no oscillations were present (Figure 7). The according absence of a direct neuronal correlate of HDO suggested that HDO may represent a physiological noise source for BOLD fMRI measurements. To evaluate the influence of this physiological noise on the analysis of fMRI data, BOLD datasets, recorded during electrical paw stimulation, were examined using two methods. First, statistical analysis was performed for calculation of BOLD activation maps using the GLM. Secondly, brain network analysis was performed using NBS. Both analyses were conducted before and after voxel-wise HDO removal using the suppression algorithm.

**FIGURE 8:**
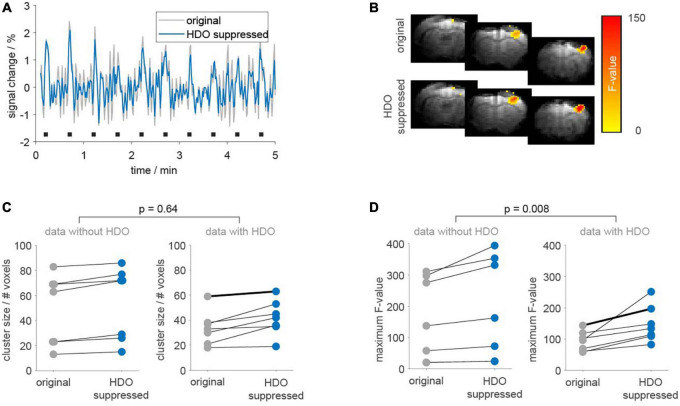
Application of the suppression algorithm improves GLM-based analysis of fMRI data. GLM-based analysis was performed on 14 fMRI datasets before (gray) and after HDO suppression (blue). **(A)** Periods (5 min) of BOLD time courses and **(B)** activation maps of one exemplary oscillating dataset. Duration of electrical paw stimulation were highlighted by black bars. **(C)** Cluster sizes and **(D)** maximum *F*-values resulting from GLM-based analysis of BOLD activation are shown separately for data with and without HDO (*n* = 7, each). Data points for identical measurements are connected by lines. Bold lines indicate the dataset shown in panels **(A,B)**. *U*-tests were used to test for significant differences between relative cluster sizes and relative *F*-values of data with and without HDO.

Statistical analysis was performed on 14 fMRI datasets (14 animals) using the GLM with the 3rd order canonical basis set. Seven datasets each were classified as oscillatory or non-oscillatory. BOLD activation maps showed larger clusters of activated voxels and larger maximum *F*-values after applying the suppression algorithm, as compared to the original data (Figure 8). Cluster sizes and maximum *F*-values obtained for HDO suppressed data were normalized to values extracted from original data. Resulting relative cluster sizes were 1.13 ± 0.08 and 1.27 ± 0.25 for non-oscillating and oscillating data, respectively. Similarly, for *F*-values, the suppression algorithm had a larger impact on oscillating data, with relative *F*-values of 1.21 ± 0.06 and 1.63 ± 0.48 for non-oscillating and oscillating data, respectively. Statistical comparisons of the normalized values (*U*-test, Bonferroni corrected) showed that the difference between oscillating and non-oscillating data was significant for normalized *F*-values (*p* = 0.008). We therefore concluded that HDO significantly affected the GLM analysis of BOLD activation.

**FIGURE 9:**
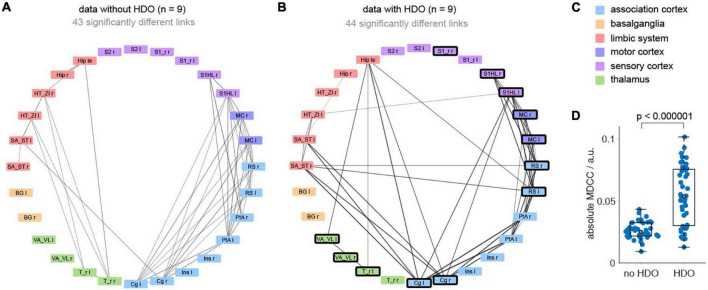
Hemodynamic oscillations elimination in fMRI data improves NBS network analysis. Brain network analysis was performed before and after application of the HDO suppression algorithm and compared using NBS. Significantly different links were displayed in circular layout, separately for **(A)** non-oscillating and **(B)** oscillating data (*n* = 9, each). Line thickness is proportional to absolute mean differences of correlation coefficients (MDCC). Nodes where HDO were frequently detected were highlighted (bold frame). **(C)** Brain regions were arranged and color coded by affiliation to six anatomical groups. **(D)** Absolute MDCC of oscillating data were significantly larger than for non-oscillating data (*U*-test).

Additionally, brain network analysis was performed on 18 GE-EPI fMRI datasets recorded in 16 animals. After data pre-processing (including lowpass filtering at 0.3 Hz and registration of a template with 28 brain regions) analysis was divided into five steps. First, nine datasets each were classified as non-oscillatory or oscillatory. In non-oscillating data sets, no oscillations were detected in any region. For oscillating data, oscillations were found in at least five brain regions. Secondly, we examined which of the brain regions showed frequent oscillations (oscillatory nodes). In total, 12 oscillatory nodes were detected, located in association cortex, motor cortex, sensory cortex, and thalamus (Supplementary Table 2). Third, for each dataset two correlation matrices were calculated (before and after the application of the HDO suppression algorithm, Supplementary Figure 2). Fourth, for both groups (oscillating and non-oscillating), correlation matrices of the HDO-unsuppressed data were compared with those of the suppressed data using NBS. Significant differences were found for both oscillating and non-oscillating data (*p* < 0.001 and *p* = 0.007, respectively, Figures 9A–C). In oscillating data, 44 correlation coefficients were significantly different. For 39 of these coefficients, at least one of the connected regions was an oscillatory node (Figures 9A, B), suggesting that differences were directly related to HDO suppression. Finally, differences in correlation coefficients were examined. Absolute MDCC of non-oscillating data were smaller when compared to oscillating data (0.03 ± 0.01 and 0.05 ± 0.02, respectively, Figure 9D). This difference was highly significant (*U*-test, *p* < 0.000001). The strong impact of the suppression algorithm on oscillatory data indicated that HDO strongly affected the network analysis of fMRI data.

## 4. Discussion

In this study, we explored the prevalence of HDO in rat fMRI measurements and their impact on analysis of fMRI data. In total 62% of all 303 fMRI measurements analyzed in this study showed HDO. This number is not a precise, general estimation of probability for the occurrence of HDO in preclinical fMRI experiments, because the measurements were conducted under widely established, specific experimental conditions, suspected to induce or inhibit HDO. However, their frequent occurrence clearly showed that HDO are relevant for functional MRI and need to be examined in more detail. For this purpose, we developed algorithms for HDO detection and suppression and evaluated fMRI measurements recorded with GE-EPI or SE-EPI sequences as well as LFP recordings.

### 4.1. Vasomotion leads to hemodynamic oscillations

We detected HDO in anesthetized rats. Several studies reported similar oscillatory signals in measurements sensitive to blood flow or arterial vessel diameter in various species such as mice (Drew et al., 2010; Mateo et al., 2017; Winder et al., 2017; Fan et al., 2020), rats (Fujii et al., 1990; Mayhew et al., 1996; Kleinfeld et al., 1998), rabbits (Hundley et al., 1988), cats (Rivadulla et al., 2011) and humans (Mitra et al., 1997; Obrig et al., 2000; Rayshubskiy et al., 2014; Liu et al., 2022), and identified vasomotion as their origin. Vasomotion refers to rhythmic oscillations of blood vessel diameter, which causes oscillations of blood flow (Aalkjaer et al., 2011). However, the physiological sources of vasomotion are still not fully understood. We also considered vasomotion as origin of the detected oscillations. Analysis of hemodynamic recordings often involves dividing signals into five frequency bands, attributed to cardiac, respiratory, myogenic, neurogenic and endothelial oscillators, respectively (Li et al., 2006; Aleksandrin et al., 2018). We divided our signals into corresponding frequency bands and found that these oscillators do not directly drive vasomotion-induced HDO. However, we cannot completely exclude that these oscillators interact with vasomotion and thus influence fMRI measurements.

### 4.2. Anesthesia exerts diverse effects on vasomotion

Most preclinical fMRI experiments use anesthesia to minimize motion and imaging artifacts. Here a well accepted anesthesia protocol was used (ISO followed by MED/DMED), for which Magnuson et al. (2010, 2014) reported the occurrence of oscillations. We intended to investigate the prevalence of these oscillations (referred as HDO) in fMRI data and identified oscillations with a frequency around 0.2 Hz in MED sedated animals. In contrast, studies applying other anesthesia or measuring awake subjects often reported a vasomotion frequency around 0.1 Hz (Fujii et al., 1990; Mayhew et al., 1996; Mitra et al., 1997; Kleinfeld et al., 1998; Obrig et al., 2000; Rivadulla et al., 2011; Rayshubskiy et al., 2014; Mateo et al., 2017; Fan et al., 2020; Liu et al., 2022). The higher oscillation frequency under MED sedation can be explained by the fact that MED is a vasoconstrictor (Sinclair, 2003; Fukuda et al., 2013) and vasoconstriction causes an increase in vasomotion frequency (Colantuoni et al., 1984; Fujii et al., 1990). In contrast to MED, ISO causes vasodilation (Flynn et al., 1992; Farber et al., 1997; Iida et al., 1998). We showed that both CO_2_ and ISO administration led to dilation of blood vessels and interrupted HDO. This is consistent with other studies showing that vasodilation decreases frequency of vasomotion, and that pronounced vasodilation abolishes vasomotion (Colantuoni et al., 1984; Fujii et al., 1990; Oude Vrielink et al., 1990; Obrig et al., 2000). We accomplished the CO_2_ challenge by replacing a portion of the oxygen in the breathing gas by CO_2_. Although the difference in oxygen supply was low (5%), we cannot explicitly assign the observed vasodilation to the resulting hypercapnia alone, a slight hypoxia may have contributed to the reduced vascular tone as well. Yet, vasodilation caused by hypercapnia can suspend vasomotion (Hudetz et al., 1998). This is relevant when using MED sedation as MED can induce hypercapnia (Brynildsen et al., 2017). However, vasomotion was not inhibited under MED sedation applied in the current study. In contrast to our results, a study in cats detected vasomotion during long-term ISO anesthesia (Rivadulla et al., 2011). However, a lower dose of ISO was used in that study, suggesting a dose-dependent effect of ISO. Accordingly, vasomotion is expected to occur not only during measurements with pure MED sedation, but also with a combination of MED and low ISO doses, a popular anesthetic regimen which provides temporally stable brain states in mice (Pradier et al., 2021), resembles the awake condition in rat brains more closely than MED or ISO alone (Paasonen et al., 2018) and enhances functional connectivity specificity as shown by Grandjean et al. (2022). Another important point is that ISO depresses sympathetic nervous activity (Skovsted and Sapthavichaikul, 1977) and that the latter was found to be a prerequisite for vasomotion (Colantuoni et al., 2001; Nilsson and Aalkjaer, 2003). Accordingly, vasomotion can only restart after end of ISO administration when both sympathetic nervous system activity has returned and vasodilation has regressed. In our experiments, the vasomotion suppressing effect of ISO had no long-term effect on consecutive measurements under MED. In contrast to Magnuson et al. (2014), we detected vasomotion already 1 h after switch from ISO to MED. Earlier times were not investigated as there was no stable physiological baseline directly after switching from ISO to MED. The early appearance of vasomotion implies that limiting experimental duration alone is unlikely to prevent the occurrence of oscillations.

### 4.3. Vasomotion significantly impacts analysis of fMRI data

Functional magnetic resonance imaging data are interpreted under the assumption that detected signal changes reflect neuronal activity due to neurovascular coupling. However, MR signal fluctuations of non-neuronal origin can distort data analysis. No direct correlation between our LFP and fMRI data was to be expected as the measurements were performed separately and LFP and hemodynamics show time-dependent variations. Nevertheless, the absence of a prominent signal component of the LFP signals at 0.2 Hz strongly suggests that the vasomotion-induced HDO have no direct neuronal correlate. This is consistent with other studies (Rivadulla et al., 2011; Winder et al., 2017). Mateo et al. (2017) reported a correlation between an envelope of γ-band (30–80 Hz) and vasomotion. In contrast to our study, these authors performed simultaneous hemodynamic and LFP recordings in awake mice. They interpreted their findings as evidence for a potential linkage between changes in brain oxygenation by modulation of vasomotion and ultra-slow variability in neuronal activity. Mechanistic insight supporting this notion is not available yet. Other studies found that vasomotion can also occur independently of neuronal activity. Winder et al. (2017) detected vasomotion after pharmacological blocking of neuronal activity, and further, spontaneous vasomotion was observed in isolated, pressurized vessels (Osol and Halpern, 1988). All together we concluded that vasomotion can introduce physiological noise in fMRI measurements and must be considered when evaluating these measurements.

While correction procedures for other physiological noise sources such as for example breathing and heart rate are available (Murphy et al., 2013), physiological noise from vasomotion is mostly neglected. Models used for GLM analysis usually ignore vasomotion and network analysis is based on the evaluation of temporal signal fluctuations irrespective of their origin. To investigate the influence of vasomotion on the analysis of fMRI data, we performed fMRI data analysis before and after HDO correction with a new suppression algorithm. Both GLM-based analysis and NBS network analysis showed that application of this algorithm had an impact on all data (measurements with and without vasomotion). However, in both analysis methods, HDO suppression had a significantly higher impact on data with vasomotion compared to vasomotion-free data. The relatively smaller effect on analysis of data without vasomotion indicated that the algorithm removed random noise. The much larger influence on data with HDO showed that vasomotion strongly affected GLM-based and NBS analysis of fMRI data.

### 4.4. Hemodynamic oscillations suppression complements preprocessing

One could argue that lowpass filtering strategies have already been implemented to minimize the impact of physiological noise in fMRI data. Complex preprocessing pipelines, such as the independent component analysis-based method FIX (Salimi-Khorshidi et al., 2014; Zerbi et al., 2015), presumably diminish the effects of vasomotion. For brain network analysis often a 0.1-Hz-lowpass filter is used for preprocessing. Indeed, its application removes HDO as effectively as the developed suppression algorithm (data not shown). However, Pan et al. (2013) showed a high correlation between LFP and fMRI ranging from 0.1 to 0.2 Hz in DMED sedated rats. Following this line of evidence, a study by Grandjean et al. (2014) set the filter to 0.3 Hz for MED/DMED anesthesia. For anesthetic regimens other than MED, we expect a lower oscillation frequency (see Section “4.2. Anesthesia exerts diverse effects on vasomotion”). Therefore, a 0.1 Hz lowpass filter may not be sufficient to remove HDO and vasomotion correction may still be needed. Accordingly, we recommend application of lowpass filter together with an HDO suppression algorithm.

### 4.5. Hemodynamic oscillations occur in both small and large blood vessels

The functional GE-EPI signal is most sensitive to large venous blood vessels, while the SE-EPI signal is most sensitive to small vessels (Buxton, 2013; Baez-Yanez et al., 2017). Since we detected oscillations in both GE-EPI and SE-EPI measurements, our results suggest that HDO occur in both small and large venous blood vessels. Since all regions of the brain are densely vascularized, oscillations are likely to occur everywhere in the brain. Therefore, it appears unlikely that it will be possible to avoid recordings of oscillations by excluding individual brain regions from the measurements. This is not only relevant for fMRI, but for all techniques that are sensitive to hemodynamic changes. In addition to optical recordings of blood volume or flow and laser doppler flowmetry, this also applies to optical recordings of fluorescent dyes, as hemodynamic changes cause artifacts in these measurements (Kozberg et al., 2016; Ma et al., 2016; Lambers et al., 2022; Zhang et al., 2022).

## 5. Conclusion

Here, we have investigated prevalence of HDO and their impact on analysis of fMRI data under anesthetic regimens that are widely used for rodent fMRI experiments. HDO were not detected under vasodilatory conditions such as for example ISO anesthesia. Under the commonly applied MED anesthesia, vasomotion-induced HDO occurred already during short experimental duration and in most regions of the brain. Since HDO significantly influenced analysis of fMRI data, HDO should be corrected during fMRI data analysis. We provide HDO detection and suppression algorithms that can be readily applied to fMRI data.

## Data availability statement

The raw data supporting the conclusions of this article will be made available by the authors, without undue reservation.

## Ethics statement

The animal study was reviewed and approved by the Landesamt für Natur, Umwelt und Verbraucherschutz of Nordrhein-Westfalen, Germany.

## Author contributions

HL and CF designed the study and wrote the manuscript. HL and LW conducted the experiments. HL and CL performed the analysis. CL developed the HDO algorithms. All authors edited and approved the manuscript.

## Footnotes

1
https://www.fil.ion.ucl.ac.uk/spm/

2
https://www.nitrc.org/frs/?group_id=889

3
https://cytoscape.org/

4
https://github.com/TheFaberLab/HDO
