# Supplementary material for: The impact of vasomotion on analysis of rodent fMRI data
Source: Front Neurosci. 2023 Feb 24;17:1064000. doi: 10.3389/fnins.2023.1064000 (PMC9998505; doi:10.3389/fnins.2023.1064000)
Supplement: Supplementary file 1 [file Data_Sheet_1.PDF]

Supplementary Material

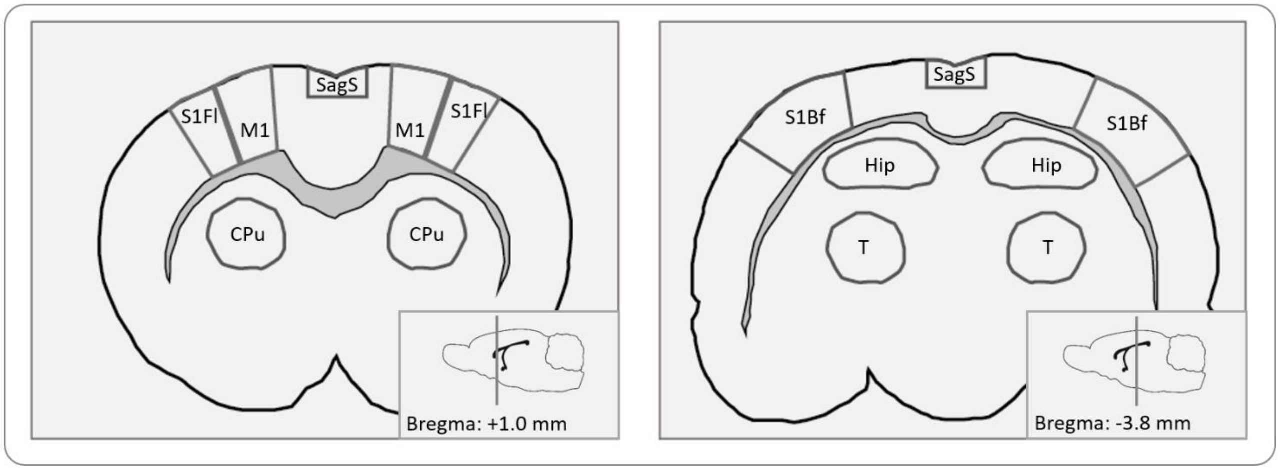

Suppl. Figure 1: Region template. fMRI time courses were extracted from 14 regions located at Bregma +1.0 mm and Bregma -3.8 mm: barrel cortex (S1Bf), Hippocampus (Hip), primary motor cortex (M1), primary somatosensory cortex, forelimb region (S1Fl), Sagittal Sinus (SagS), Striatum (CPu), Thalamus (T).

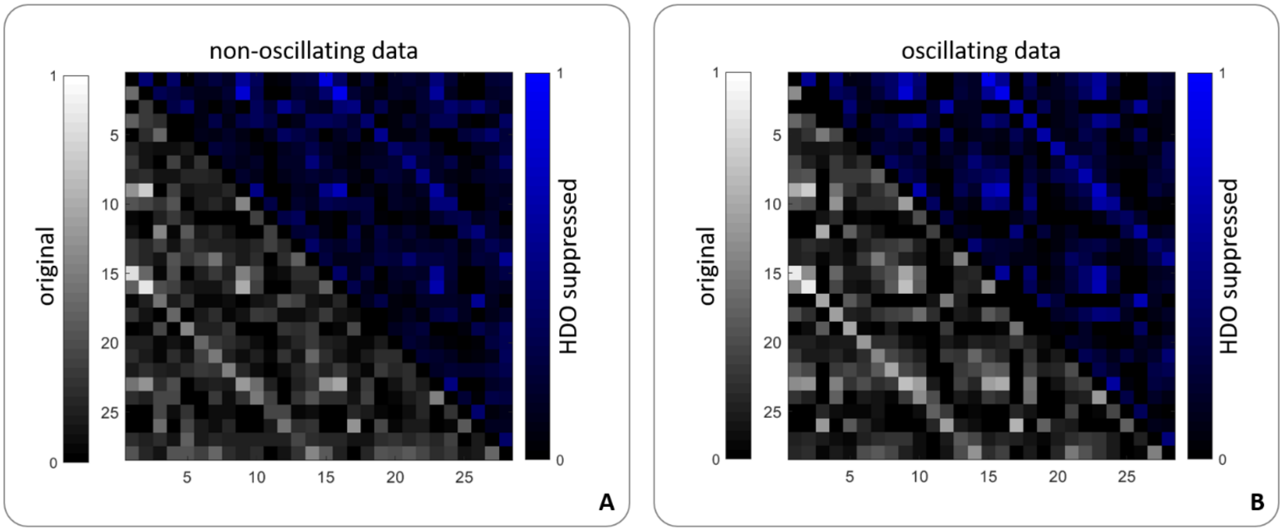

Suppl. Figure 2: Average group connectivity matrices. Correlation coefficients of 18 GE-EPI datasets were calculated between pairs of 28 brain regions before (grey) and after (blue) application of the hemodynamic oscillation (HDO) suppression algorithm. Resulting correlation matrices, representing weighted functional connections were averaged for (A) non-oscillation and (B) oscillation datasets (n = 9, each). Only positive correlation coefficients were considered. Diagonals, representing interhemispheric connections between regions are clearly visible, indicating that the analysis yielded meaningful functional connections despite the anatomical distance.

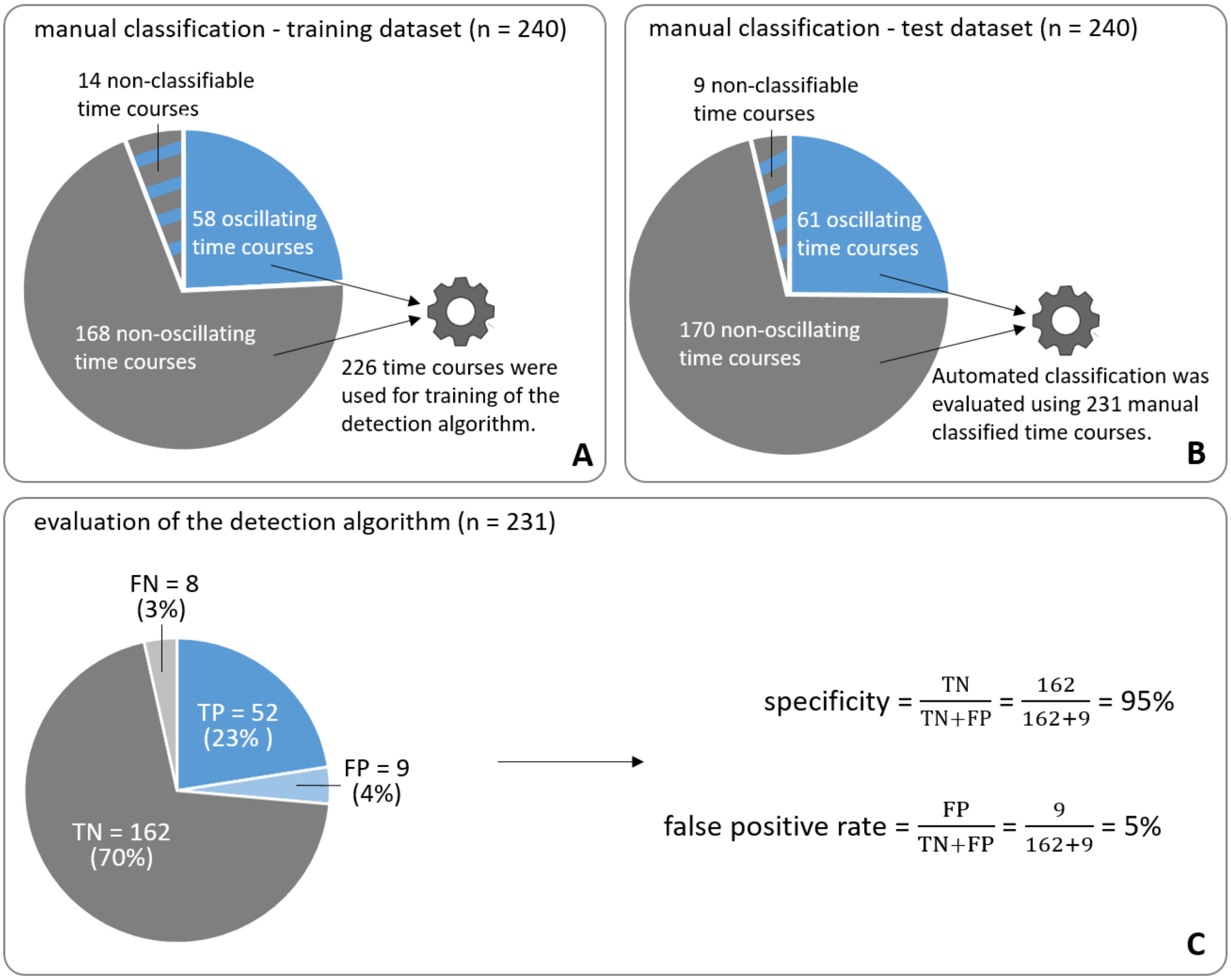

Suppl. Figure 3: Training and test datasets. For both training and test data, 240 time courses each were randomly selected from 2254 time courses of high temporal resolution GE-EPI data (Suppl. Table 1). Training and evaluation of the detection algorithm was done using manual classifications of (A) training data and (B) test data, separating oscillating from non-oscillating time courses. It should be noted that for training and test data 226 and 231 time courses, respectively, could be classified unambiguously. Training of the detection algorithm was done using the 226 classified training time courses. Subsequently, the performance of the algorithm was evaluated using the 231 manually classified time courses of the test dataset: Based on the manual classification, the results of the detection algorithm were subdivided into true positive (TP), false positive (FP), true negative (TN) and false negative (FN). The detection algorithm had a specificity of 95% with a false positive rate of 5%.

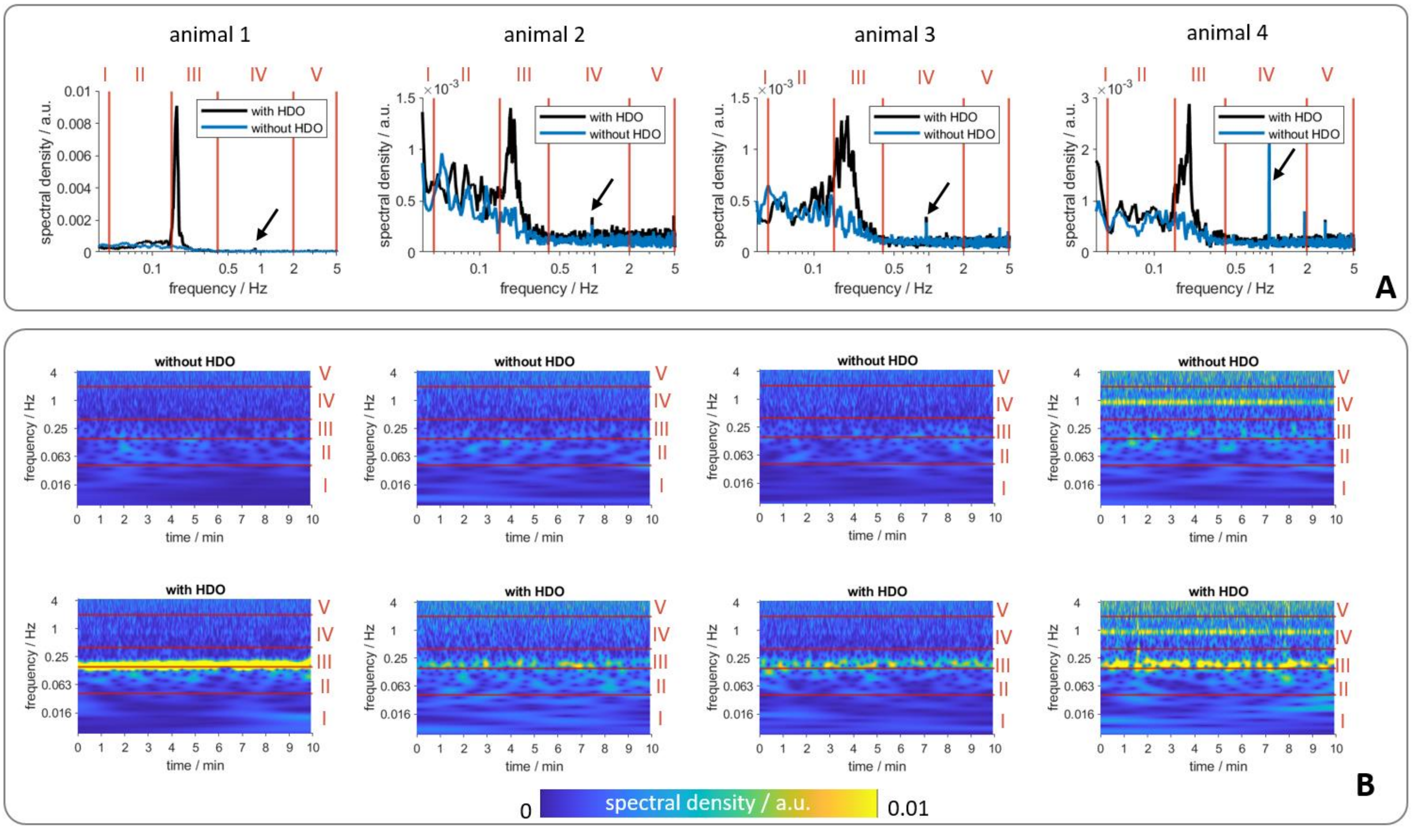

Suppl. Figure 4: Hemodynamic oscillations (HDO) are not dominated by cardiac, respiratory, myogenic, neurogenic and endothelial oscillators. Assessment of frequency domain of exemplary fMRI time courses recorded for 10 minutes in S1Fl of four animals under resting state conditions with a TR of 100 ms. Per animal, frequency domain of one measurement with and one without HDO was analyzed using MATLAB. Red lines mark 5 frequency bands which correspond to (I) endothelial, (II) neurogenic, (III) myogenic, (IV) respiratory and (V) cardiac activities, respectively. (A) Fast Fourier Transformation (FFT) of time courses without HDO (blue) and with HDO (black) showed no substantial differences except for the peak at 0.2 Hz caused by HDO. All spectra showed a peak around 1 Hz caused by respiration (arrow). As animals were ventilated, this peak was narrow. In animal four, the respiration peak had a large amplitude and higher harmonics around 2 Hz and 4 Hz, indicating strong respiratory motion artifacts. Logarithmic x-axis range from 0.03 Hz to 5 Hz and spectra were smoothed with a span of 5 data points. (B) Temporal trends of the frequency bands were displayed using continuous wavelet transformation (CWT). For each frequency, the spectral density was color-coded and plotted versus time. Both HDO and respiration are visible at 0.2 Hz and approximately 1 Hz, respectively. Similar to FFT, CWT showed only one difference between data without and with HDO: the occurrence of an oscillation at 0.2 Hz. The investigated frequency bands did not differ substantially for data with and without HDO within one animal. In summary, both FFT and CWT showed that the peak at 0.2 Hz caused by HDO was the only conspicuous change in frequency domain when HDO occurred.

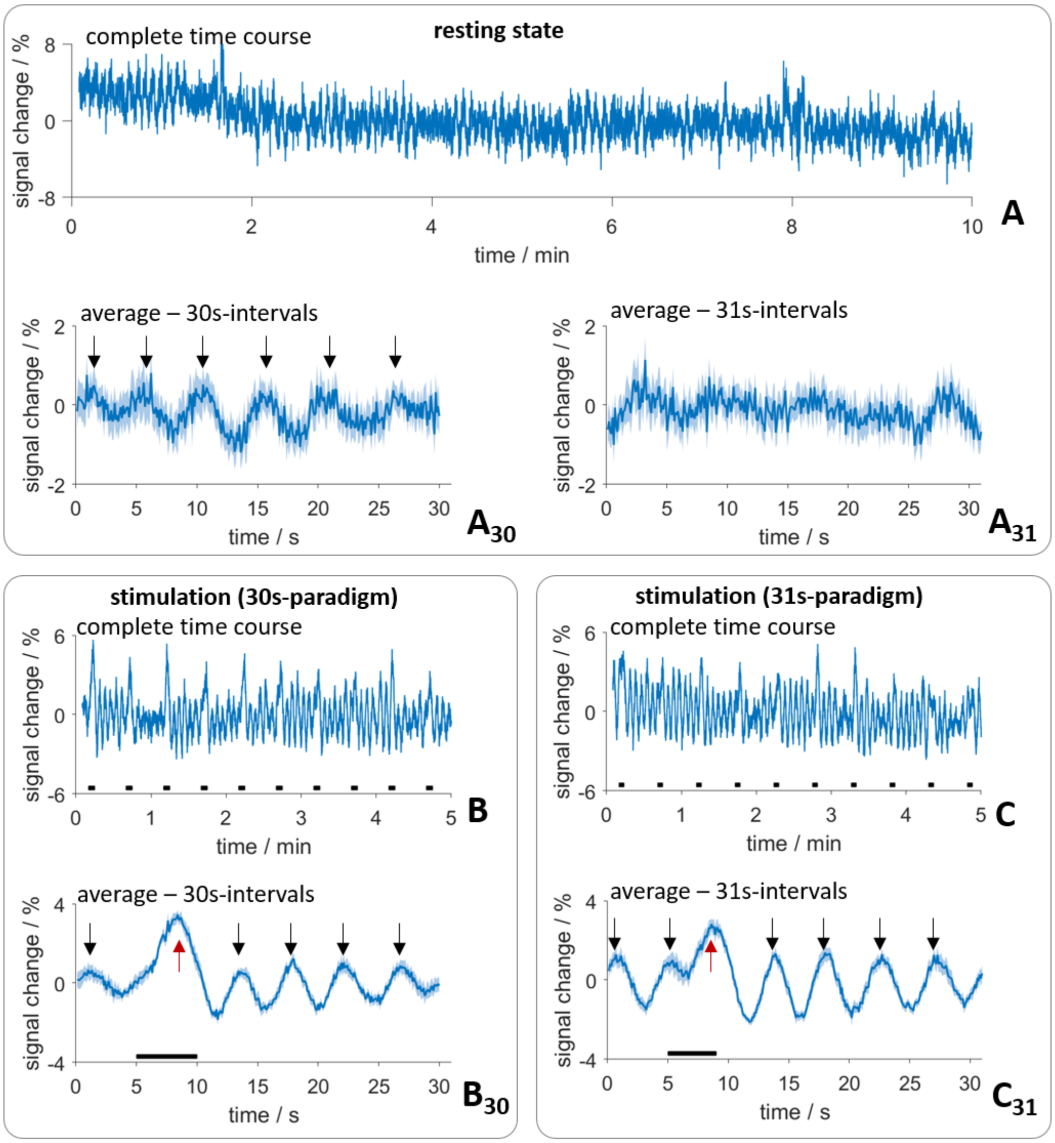

Suppl. Figure 5: Hemodynamic oscillations (HDO) are phase-locked to stimulation of neuronal activation. Exemplary time courses with HDO recorded in S1Fl of MED sedated animals at TR = 100 ms. In addition to the complete time courses (upper row), averaged signals are shown (lower row, mean ± standard error of the mean). To calculate averaged signals, time courses were divided into either 30 s or 31 s long intervals (indicated by subscript), and these intervals were averaged. (A) Resting state measurement: The resting state fMRI signal averaged over 30 s long intervals (A_{30}) showed an oscillating signal with 6 peaks (black arrows), while the fMRI signal averaged over 31 s long intervals (A_{31}) showed no clear periodicity. We conclude that without stimulation, HDO are averaged out if the averaging interval length is not equal to a multiple of HDO period (5 s). (B, C) Measurements recorded during electrical paw stimulation (9 Hz, 1.5 mA, 1 ms pulse length, stimulation periods are indicated by black bars) with (B) a 30 s long paradigm (5 s stimulation, 25 s rest) and (C) a 31 s long paradigm (4 s stimulation, 27 s rest). Both paradigms were applied in block design with 10 repetitions. (B_{30}, C_{31}) For stimulus-evoked BOLD measurements, HDO (maxima indicated by black arrows) were conserved when the averaging interval coincided with the paradigm length, for both 30 s and 31 s. It is also clearly

visible that the BOLD response (red arrows) interrupted HDO, and HDO restarted in a phase-locked manner after the BOLD response.

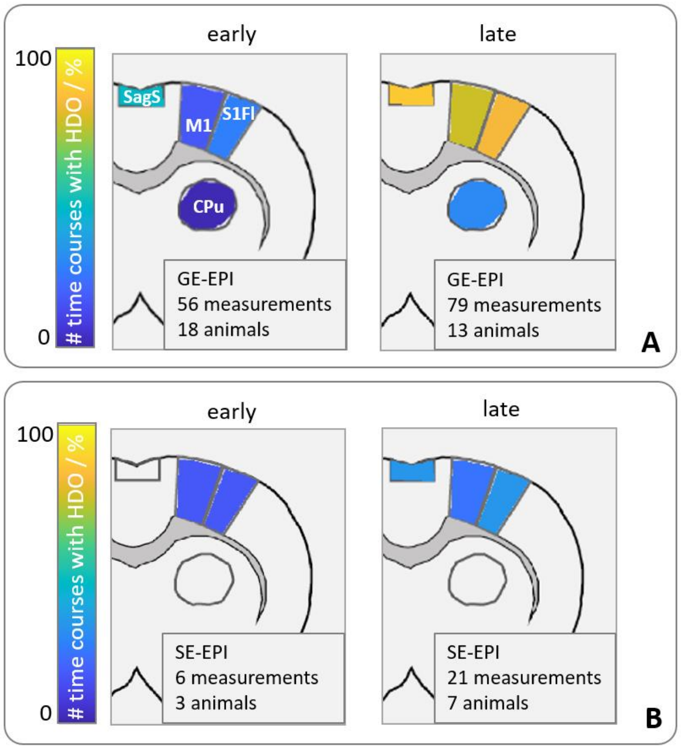

Suppl. Figure 6: HDO prevalence maps for (A) GE-EPI and (B) SE-EPI data. Maps were subdivided into early (recorded 1-3 h after MED start) and late (recorded 4 h or later) measurements. Color encodes relative number of scans with HDO in 5 regions (primary motor cortex (M1), primary somatosensory cortex, forelimb region (S1Fl), Sagittal Sinus (SagS), Striatum (CPu)) located in the right hemisphere at Bregma +1.0 mm. HDO-free regions are not colored. In GE-EPI measurements, HDO occurred in all investigated brain regions and HDO prevalence increased over time. In contrast, in SE-EPI data HDO were observed only in cortical regions and HDO prevalence increased only slightly over time. These differences can be partly attributed to the fact that tSNR of GE-EPI data was about three times higher than tSNR of SE-EPI data. Detection of HDO in SE-EPI data, therefore, requires a higher amplitude compared to GE-EPI measurements.

Supplementary Table 1: Overview of conducted experiments (continued on page 7).

data group	experiment	examinations	remarks	animals / # [a]	Scans /#	time courses / #	regions	sequence	TR / ms	duration / min
ISO & GE-EPI	1) long-term ISO anesthesia: Scans were recorded repeatedly for up to 4 h under isoflurane anesthesia.	effect of long-term vasodilation	/	♀: 10<br>♂: 0	56	784	CPu, Hip, M1, S1Bf, S1Fl, SagS, T	GE-EPI	125	5 or 10
MED & GE-EPI	2) Starting at least 40 min after switching to MED sedation, scans were recorded repeatedly for up to 8 h.	HDO prevalence under MED sedation in GE-EPI data	In 10 animals, long-term ISO scans were performed previously and MED scans were maximal conducted for 2 h. In 5 animals, scans did not start until 4 h after switching anesthesia.	♀: 22<br>♂: 1	135	1232	CPu, Hip, M1, S1Bf, S1Fl, SagS, T	GE-EPI	100 or 125	5 or 10
MED & SE-EPI	3) SE-EPI scans were recorded 2-8 h after starting MED.	HDO prevalence under MED sedation in SE-EPI data	In one animal 1 extra GE-EPI scan was taken (TE = 35 ms, TR =250 ms) for direct comparison of GE- & SE-EPI.	♀: 7<br>♂: 0	27	189	CPu, M1, S1Fl, SagS	SE-EPI	250	10
CO_2 challenge	4) CO_2 was added to inspiratory air. Scans were performed 1-4 h after starting MED.	effect of vasodilation challenges	/	♀: 13<br>♂: 0	15	15	S1Fl	GE-EPI	1000	10
ISO challenge	5) ISO was administered for 10 minutes. Scans were performed 1-4 h after starting MED.	effect of vasodilation challenges	/	♀: 14<br>♂: 0	20	20	S1Fl	GE-EPI	1000	25

[a] In some animals several experiments were conducted. Numbers were given separately for females (♀) and males (♂).

Supplementary Table 1 (continued)

data group	experiment	examinations	remarks	animals / # [a]	Scans /#	time courses / #	regions	sequence	TR / ms	duration / min
sensory stimulation	6) Data were recorded during electrical paw stimulation applied in block paradigm.	A) impact of HDO to GLM-based analysis B) impact of HDO to NBS analysis	A) Scans with <13 or >100 activated voxels were excluded. 2 scans were omitted due to disagreement with the detection tool. B) Non-registerable data were excluded.	♀: 27 ♂: 0	31	n.s.	A) S1Fl B) see Suppl. Table 2	GE-EPI	1000	10
LFP recordings	7) LFP recordings were conducted 1-4 h after starting MED sedation.	search for neuronal correlate	/	♀: 6 ♂: 0	32	32	S1Fl	/	/	/
only in training/test data	Data were recorded under ISO anesthesia, MED sedation or during the change of anesthesia.	Excluded from further examinations as data were recorded during the change of anesthesia or scans were wrong positioned.	/	♀: 7 ♂: 1	18	238	CPu, Hip, M1, S1Bf, S1Fl, SagS, T	GE-EPI	100 or 125	5 or 10
training data	randomly selected from GE-EPI scans, recorded with TR = 100 or 125 ms	A) HDO frequency characterization B) training of the ANN implemented in the detection algorithm	14 time courses were not manually classifiable and were not used for training of the detection algorithm.	♀: 19 ♂: 1	110	240	CPu, Hip, M1, S1Bf, S1Fl, SagS, T	GE-EPI	100 or 125	5 or 10
test data	randomly selected from GE-EPI scans, recorded with TR = 100 or 125 ms	evaluation of the HDO detection algorithm	9 time courses were not manually classifiable and were not used for evaluation of the detection algorithm.	♀: 22 ♂: 1	145	240	CPu, Hip, M1, S1Bf, S1Fl, SagS, T	GE-EPI	100 or 125	5 or 10

[a] In some animals several experiments were conducted. Numbers were given separately for females (♀) and males (♂).

Supplementary Table 2: Brain regions used for network analysis. Regions in which at least five oscillatory time courses were detected were classified as oscillatory nodes.

anatomical group	region	hemisphere	abbreviation	non-oscillating data HDO time courses / #	oscillating data HDO time courses / #	oscillatory node
association cortex	retrosplenial cortex	left	RS l	0	9	yes
association cortex	retrosplenial cortex	right	RS r	0	9	yes
association cortex	cingulate cortex	left	Cg l	0	9	yes
association cortex	cingulate cortex	right	Cg r	0	9	yes
association cortex	insular cortex	left	Ins l	0	2	no
association cortex	insular cortex	right	Ins r	0	3	no
association cortex	parietal association cortex	left	PtA l	0	4	no
association cortex	parietal association cortex	right	PtA r	0	4	no
basalganglia	basalganglia	left	BG l	0	2	no
basalganglia	basalganglia	right	BG r	0	2	no
limbic system	hypothalamus + zona incerta	left	HT_ZI l	0	2	no
limbic system	hypothalamus	right	HT_ZI r	0	3	no
limbic system	hippocampus	left	Hip le	0	2	no
limbic system	hippocampus	right	Hip r	0	3	no
limbic system	septal area + bed nucleus of stria terminalis	left	SA_ST l	0	3	no
limbic system	septal area + bed nucleus of stria terminalis	right	SA_ST r	0	2	no
motor cortex	motor cortex	left	MC l	0	8	yes
motor cortex	motor cortex	right	MC r	0	6	yes
sensory cortex	primary somatosensory cortex hind limb	left	S1HL l	0	5	yes
sensory cortex	primary somatosensory cortex hind limb	right	S1HL r	0	7	yes
sensory cortex	primary somatosensory cortex rest	left	S1_r l	0	4	no
sensory cortex	primary somatosensory cortex rest	right	S1_r r	0	5	yes
sensory cortex	secondary somatosensory cortex	left	S2 l	0	4	no
sensory cortex	secondary somatosensory cortex	right	S2 r	0	3	no
thalamus	thalamic nucleus ventralis anterolateralis	left	VA_VL l	0	6	yes
thalamus	thalamic nucleus ventralis anterolateralis	right	VA_VL r	0	5	yes
thalamus	thalamus rest	left	T_r l	0	6	yes
thalamus	thalamus rest	right	T_r r	0	4	no
